# Zeptojoule calorimetry

**DOI:** 10.1038/s41928-026-01615-2

**Published:** 2026-05-12

**Authors:** András Márton Gunyhó, Kassius Kohvakka, Qi-Ming Chen, Jean-Philippe Girard, Roope Kokkoniemi, Wei Liu, Mikko Möttönen

**Affiliations:** 1 QCD Laboratories, QTF Centre of Excellence, Department of Applied Physics, Aalto University, Espoo, Finland; 2 IQM Quantum Computers, Espoo, Finland; 3 QTF Centre of Excellence, VTT Technical Research Centre of Finland Ltd, Espoo, Finland

**Keywords:** Single photons and quantum effects, Superconducting devices, Electrical and electronic engineering, Sensors

## Abstract

The measurement of energy is a fundamental tool used in quantum technology and computing. Some of the most sensitive energy detectors—bolometers and calorimeters—are thermal, meaning that they operate by absorbing incoming energy, converting it into heat and reading out the resulting temperature change electrically using a thermometer. Recently, superconductor–normal-conductor–superconductor radiation sensors with metallic and graphene absorbers haven been predicted to be capable of full-width at half-maximum energy resolutions of 0.75 zJ and 0.05 zJ, respectively. However, these estimates are only mathematically extracted from steady-state noise and responsivity measurements. Here we show that a metallic superconductor–normal-conductor–superconductor sensor can be used for zeptojoule calorimetry. With the approach, we measure the energy of 1-μs-long 8.4-GHz microwave pulses with a full-width at half-maximum energy resolution finer than 0.95 ± 0.02 zJ (=5.9 ± 0.12 meV) corresponding to 170 photons at 8.4 GHz. The technique provides a potential path to real-time calorimetric detection of single photons in the 10-GHz range.

## Abstract (web summary)

A metallic superconductor–normal-conductor–superconductor sensor can be used to measure the energy of 1-μs-long 8.4-GHz microwave pulses with a full-width at half-maximum energy resolution finer than 0.95 ± 0.02 zJ, corresponding to 170 photons at 8.4 GHz.

## Main

The detection of weak electromagnetic signals is important in a range of scientific and practical applications, including fundamental explorations of quantum thermodynamics^1,2^, the search for axions^3,4^, as well as quantum technology and computing^5–7^. This has led to the development of several kinds of sensitive radiation sensors operating at cryogenic temperatures. At present, it is possible to detect individual microwave photons below 10 GHz using superconducting qubits^8,9^, current-biased Josephson junctions^10–12^ and nonlinear oscillators^13^. However, such detectors are usually not energy resolving, and they may suffer from narrow bandwidths for the signal to be detected, typically on the order of a few megahertz.

Detecting single photons in an energy-resolving manner over a broad frequency band is possible using sensors where the incoming photons break Cooper pairs in a superconductor, which is the basis of kinetic inductance detectors^14,15^, superconducting nanowire single-photon detectors^16,17^ and quantum capacitance detectors^18,19^. Recently, single 25-μm (7.95 zJ) photons have been resolved using a kinetic inductance detector^20^, while single-photon detection down to energies of 1.5 THz × *h* ≈ 0.99 zJ has been demonstrated using quantum capacitance detectors^18,19^.

The fundamental limit of the energy measured by pair-breaking detectors is set by the energy gap of the absorber material, which is roughly 100 GHz × *h* for Al for example. On the other hand, thermal detectors^21^, such as magnetic microcalorimeters^22,23^, transition-edge sensors^24,25^ and graphene-based devices^26,27^, are only limited by thermal fluctuations due to their coupling to a heat bath^28^. This has been predicted to enable energy resolutions as fine as 2 GHz × *h* (ref. ^29^). The most sensitive calorimeters reported to date are graphene-based devices, which are able to detect single 1,550-nm (193 THz × *h* = 128 zJ) photons^30^, and titanium transition edge sensors, which have been shown to have a resolution of 49 THz × *h* = 32.5 zJ for 20-GHz photons^31^ and 26.6 THz × *h* = 17.6 zJ for 8-μm photons^32^. However, calorimetry in the microwave regime (<300 GHz), relevant, for example, for the axion search, remains a challenge.

Recently, superconductor–normal-conductor–superconductor (SNS) radiation sensors with metallic^33^ and graphene^34,35^ absorbers have demonstrated noise equivalent powers (NEPs) on the order of tens of ${\rm{zW}}/\sqrt{{\rm{Hz}}}$ when they were operated as continuous power meters, that is, as bolometers. On the basis of these NEP values, it was predicted that these sensors should have full-width at half-maximum (FWHM) energy resolutions of 0.75 zJ and 0.05 zJ = 71 GHz × *h* for the metallic and graphene absorber, respectively.

In this Article, we show that a metallic SNS sensor^33,36^ can be used as a zeptojoule calorimeter. We first characterize the NEP of the sensor in the bolometric mode, and subsequently record individual traces of the detector signal in the time domain while applying short microwave pulses in the zeptojoule regime at the input of the sensor. By using a matched filter on the traces, we find a FWHM energy resolution finer than 0.95 zJ ≈ 1.4 THz × *h*. Our approach allows photon counting in the terahertz regime and provides a step towards the calorimetric detection of microwave photons.

## Device operation

**Fig. 1:**
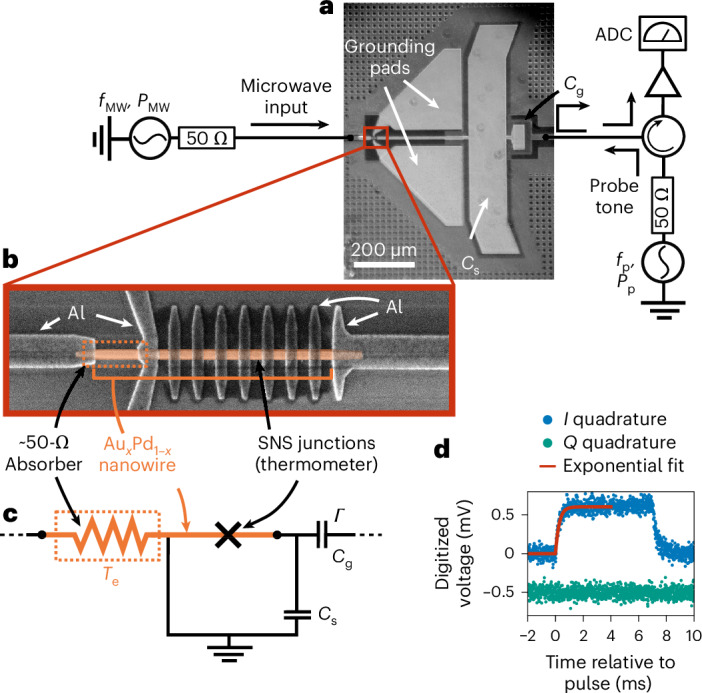
SNS radiation sensor and its operating principle. **a**, Optical microscope image of the SNS sensor chip together with a simplified measurement setup. **b**, False-colour scanning-electron micrograph of a nominally identical chip showing the inherently non-superconducting AuPd nanowire (orange) and the SNS junctions formed by the short segments of the nanowire between Al islands placed on top of it. **c**, Equivalent circuit diagram of the absorber and probe circuit of the sensor. The segment highlighted in orange corresponds to the nanowire that has electron temperature *T*_e_. **d**, Example of response of the sensor on absorption of a 7-ms-long 8.40 GHz microwave pulse. The two quadratures of the digitized probe signal are shown (blue and green dots), as well as an exponential fit to the rising edge (red curve), used to extract the thermal time constant. The quadrature-phase trace is offset by −0.5 mV for clarity. ADC, analogue-to-digital conversion.

Our SNS radiation sensor, similar to the device discussed in ref. ^33^, consists of an approximately 150-nm wide and 30-nm thick AuPd normal-metal nanowire, which is split into two segments, as shown in Fig. 1a–c. The first segment, around 1 μm in length, acts as the absorber, which is essentially a resistor that is impedance-matched with the input microwave transmission line. The thermometer part consists of several superconducting Al islands placed on top of the nanowire. Due to the proximity effect^37,38^, the superconducting order parameter of Al penetrates into the nanowire and hence this structure essentially constitutes SNS Josephson junctions connected in series^37^. The inductance of the junctions changes with the electron temperature *T*_e_ of the absorber, as discussed below. We capacitively shunt the SNS junctions by a 134-pF capacitor *C*_s_, which in combination with the Josephson inductance of the junctions forms an inductor-capacitor (LC) oscillator. The absorber and thermometer segments are separated by placing a superconducting Al lead that is shorted to ground on top of the nanowire. This effectively isolates them electrically, while maintaining strong thermal coupling. For details of the device fabrication, see refs. ^39,40^.

When absorbing microwave photons, the incoming energy excites quasiparticles in the absorber segment and results in a new quasiequilibrium temperature across the whole nanowire on a timescale of nanoseconds^41,42^. This temperature rise decreases the critical current and increases the Josephson inductance of the short junctions on the thermometer side, which induces a shift of the resonance frequency of the LC oscillator. This shift is read out by reflecting a probe tone of frequency *f*_p_ and power *P*_p_ off of the gate capacitor *C*_g_, and by observing changes in the reflection coefficient *Γ* near the resonance frequency.

**Extended Data Fig. 1:**
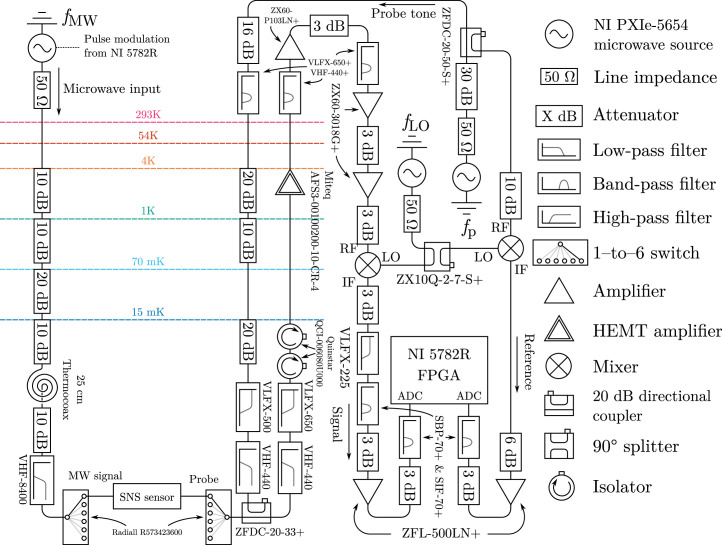
Full experimental setup. Labels below components denote Mini-Circuits model numbers. The band-pass filters consist of a low-pass filter and high-pass filter in series. The NI 5782R FPGA board handles triggering the microwave pulse via pulse modulation, as well as analog-to-digital conversion (ADC) and data acquisition. A control computer (not shown) is used to initiate each measurement and collect the data.

The device is cooled down to 20 mK in a dilution refrigerator. In both the bolometric and calorimetric operating modes, a continuous probe tone of frequency *f*_p_ ≈ 600 MHz is reflected off of the device, and subsequently filtered, amplified and digitized at room temperature in a heterodyne configuration (see Extended Data Fig. 1 and Methods for the full experimental setup). An example of the recorded time-domain signal is shown in Fig. 1d, where a microwave pulse with length *t*_MW_ = 7 ms, frequency *f*_MW_ = 8.40 GHz and power *P*_MW_ = −149.2 dBm at the chip input is applied, and the signal is averaged over 2^13^ repetitions. Here a phase rotation has been applied to the in-phase (*I*) and quadrature-phase (*Q*) heterodyne components of the data such that the signal lies almost entirely in the *I* component. The quasistatic responsivity *δV*/*δP*_MW_ is obtained from the difference between the signal during and before the pulse, whereas the thermal time constant *τ* is extracted from the exponentially rising edge of the signal at the arrival of the pulse (Methods).

## Noise characterization

We first characterize the NEP of the sensor, which allows estimating the energy resolution (Methods). We apply an effectively constant (*t*_MW_ ≫ *τ*) microwave input field, with the input power set to *P*_MW_ = −149.2 dBm to minimize the nonlinearity of the quasistatic response. The signal is averaged over 2^13^ repetitions to ensure a sufficient signal-to-noise ratio (SNR).

**Fig. 2:**
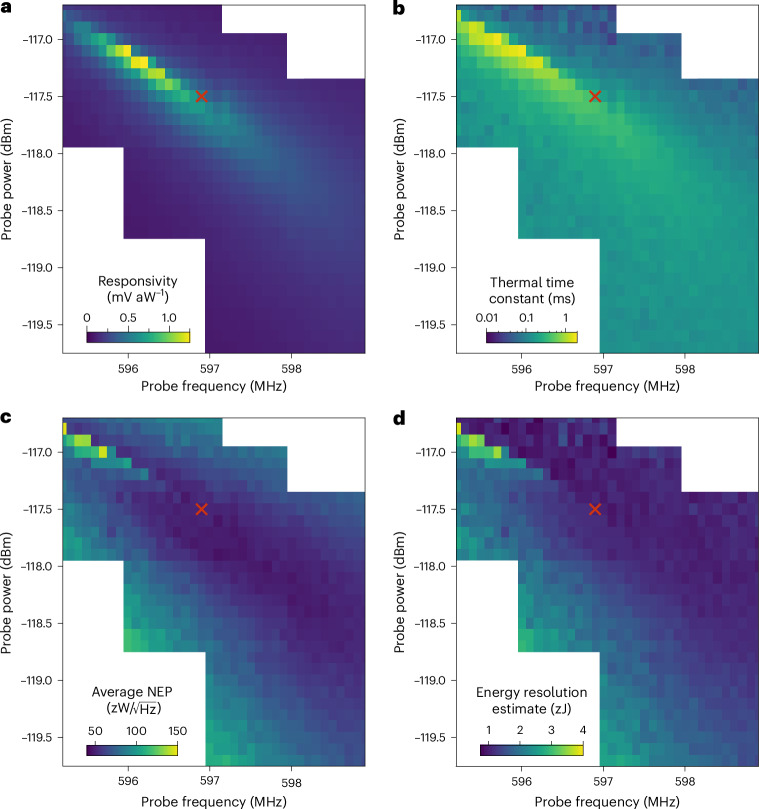
Characterization results of the SNS sensor. **a**, Quasistatic responsivity of the sensor as a function of probe frequency *f*_p_ and power *P*_p_. The responsivity is measured with a microwave signal power of −149.2 dBm. The red cross marks the operation point at *f*_p_ = 596.9 MHz, *P*_p_ = −117.5 dBm chosen for the calorimetry. **b**, Rising-edge thermal time constant *τ* extracted from the time traces of the responsivity measurement by an exponential fit. **c**, NEP of the sensor averaged over 300 Hz–1 kHz, as a function of probe frequency and power. **d**, Theoretical estimate for the FWHM energy resolution computed from the NEP data. See main text for more details.

The probe frequency *f*_p_ and power *P*_p_ are varied to find an optimal working point, see Fig. 2. The responsivity (Fig. 2a) is extracted from time-domain data similar to those shown Fig. 1d. The responsivity reaches its highest values slightly below the resonance frequency, where a change in the resonance frequency causes a large change in the transmission. Furthermore, the measured signal is proportional to the square root of the probe power, and thus increasing *P*_p_ increases the responsivity. However, a high probe power also exhibits Josephson nonlinearity and electrothermal feedback^39,21^, owing to self-heating of the nanowire. This causes a shift in the resonance frequency, and the consequent shift in the maximum of the responsivity visible in Fig. 2a. Simultaneously, the shift induces a sharp increase in the thermal time constant, shown in Fig. 2b, even with a fixed microwave power *P*_MW_. The optimal probe parameters for the calorimetry are thus a trade-off between the responsivity and the time constant. At excessively high probe powers, the feedback causes bistability in the temperature of the nanowire, which can be used for highly sensitive threshold detection^39^, at the cost of substantial dead time between measurements. However, we avoid this bistable region of the probe parameters, to operate our device as a calorimeter where the signal is proportional to the energy of the absorbed pulse, as opposed to a binary-valued click detector.

**Extended Data Fig. 2:**
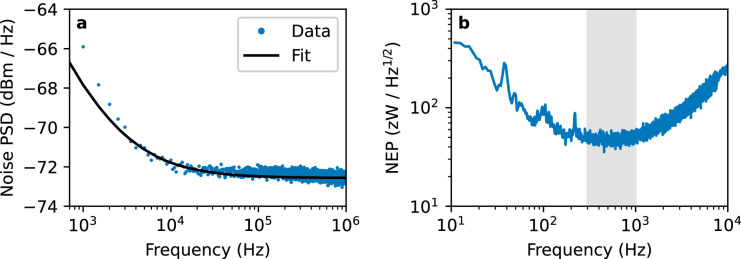
Noise power spectral density (PSD) and noise equivalent power (NEP) at the operation point. **a**, Noise power spectral density as a function of noise frequency (points), and a fit to the heuristic model *A*/*f* + *B* (solid line). **b**, Noise equivalent power as a function of noise frequency. The shaded region indicates the range over which the NEP is averaged to obtain the data in Fig. 2c.

To obtain the NEP, which is defined as the square root of the noise power spectral density (PSD) in the probe signal in units of the input microwave power, we also measure the noise PSD of the probe signal at each *f*_p_ and *P*_p_ (Methods and Extended Data Fig. 2). Figure 2c shows the resulting NEP averaged over the frequency range 300 Hz–1 kHz. An NEP below $50\,{\rm{zW}}/\sqrt{{\rm{Hz}}}$ is reached between probe powers of *P*_p_ = −118.5 and −117.5 dBm. This is on par with previous record results achieved without a parametric amplifier at the millikelvin stage^33^.

From the measured frequency-dependent NEP, we estimate the corresponding calorimetric energy resolution of the sensor using equation (6) in the Methods and show the results in Fig. 2d. Note that the finest energy resolution does not exactly coincide with the lowest average NEP, owing to the fact that the energy resolution is integrated from 0 MHz up to 31.25 MHz, the maximum sampling frequency we used to measure the noise spectrum, in contrast to the 700 Hz averaging range used for the NEP. The finest estimated energy resolution is 0.74 zJ at *P*_p_ = −117.0 dBm, *f*_p_ = 597.1 MHz. However, at powers above −117.5 dBm, the bistability may be substantial, and hence we choose the operation point with *P*_p_ = −117.5, where *f*_p_ = 596.9 MHz yields the lowest NEP. At these probe parameters, we find a tank circuit resonance frequency of 595.9 MHz and a linewidth of *γ*/(2*π*) = 4.8 MHz when no microwave signal is applied, *τ* = 260 μs, average ${\rm{NEP}}=49\,{\rm{zW}}/\sqrt{{\rm{Hz}}}$ and an estimated energy resolution of 1.03 zJ.

## Calorimetric measurements

Having found a promising operation point, we move to calorimetric measurements to measure the energy resolution instead of merely extracting it from the NEP. Here we measure probe signal traces with no ensemble-averaging, while sending 1 μs pulses, much shorter than the thermal time constant *τ*, at 8.40 GHz to the calorimeter input, with pulse energies ranging between 0.95 zJ and 3.8 zJ (≈170–680 photons). The pulse energies are accurately known since the input line attenuation has been carefully calibrated (Methods).

**Fig. 3:**
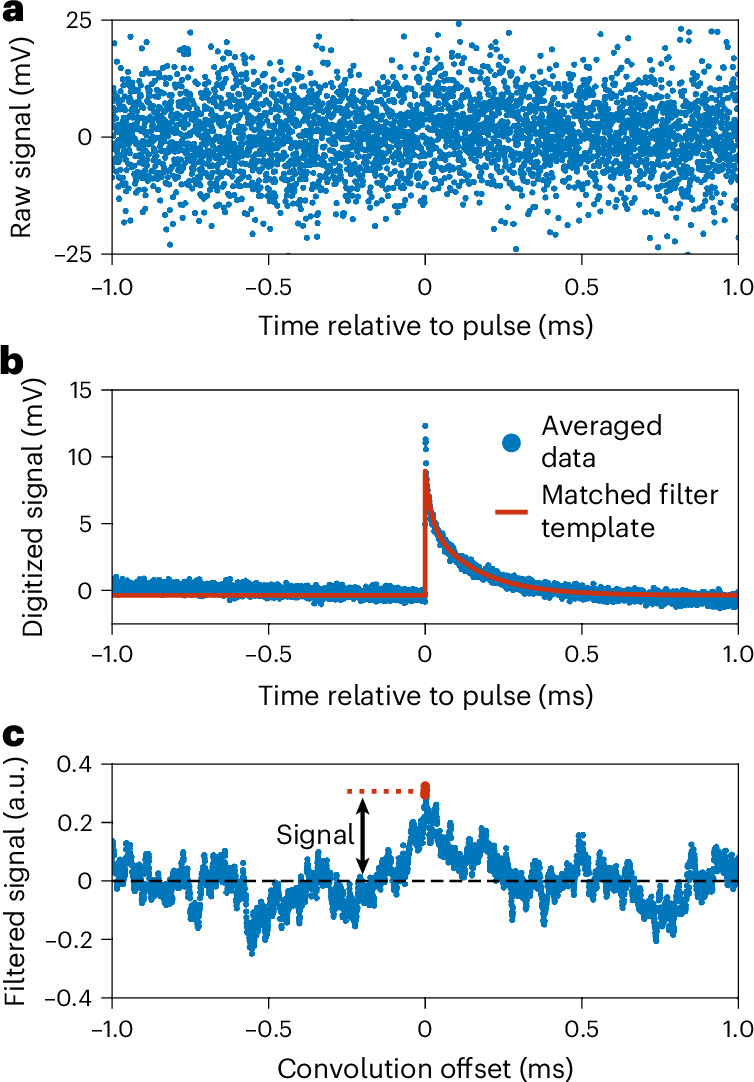
Matched-filtering procedure. **a**, Raw digitized time trace of the probe signal in a single-shot measurement, with a 1-μs calorimeter input pulse having a mean energy of 1.19 zJ applied at *t* = 0. **b**, As **a** but for a 1-μs input pulse having energy of approximately 3.8 zJ averaged over 1,000 repetitions (blue dots). A fit to a model that is a sum of two exponential decays is also shown (red curve). The model is used as the template of the matched filter (Methods). **c**, Data of **a** after application of the matched filter according to the fitted template in **b**, as a function of the convolution offset between the template and the data. The calorimetric signal $\bar{S}$ is obtained as the value of the filtered signal averaged over a 1-μs range around zero offset, highlighted in red.

Such low pulse energies result in an extremely low SNR in the raw probe signal traces, as shown in Fig. 3a. Consequently, we use a matched filter, a commonly used method in calorimetry^21,43^, which greatly improves the SNR. To extract a template for the matched filter, we average 1,000 pulses with 3.8 zJ of energy and fit a model for the expected temporal envelope of the signal (Fig. 3b and Methods). Figure 3c shows the output of the matched filter, as a function of the offset of the convolution between the transfer function of the filter and the input data. We average over a 1 μs window around the zero convolution offset to obtain the calorimetric signal $\bar{S}$. For comparison, we also carry out a simple averaging of the non-matched-filtered data over varying time windows. We find that for the 0.95 zJ pulse, the SNR from matched filtering is approximately 30% higher than the highest SNR with simple averaging, which is achieved with a 27-μs window.

**Fig. 4:**
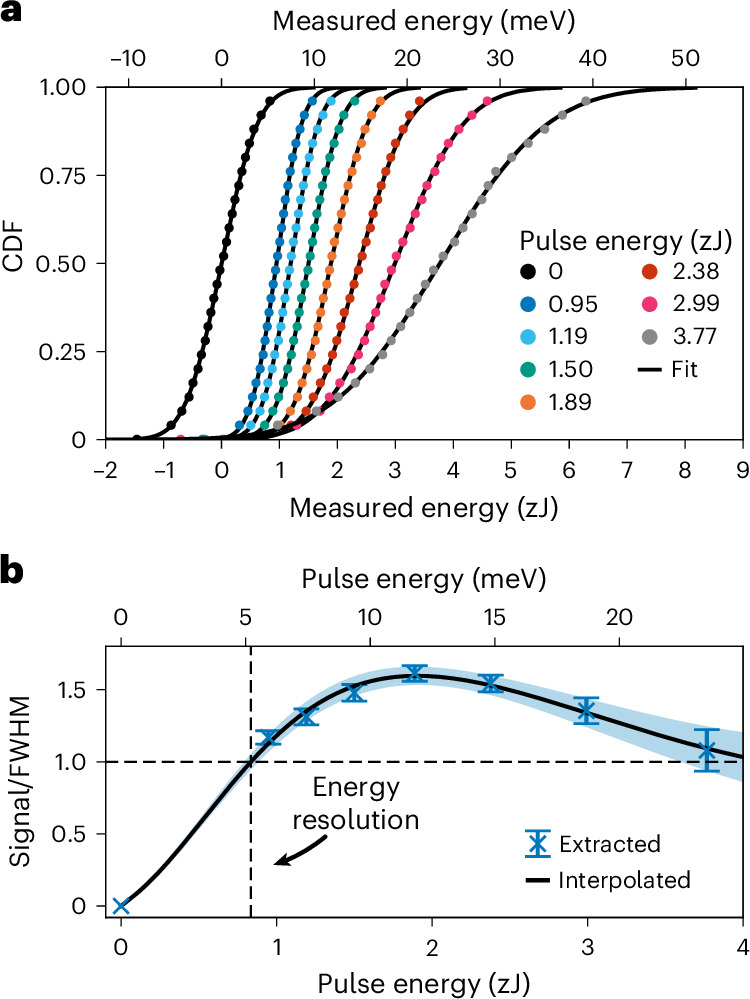
Zeptojoule calorimetry. **a**, Empirical CDF of the calorimetric signal converted to energy units as a function of pulse energy (dots). The CDF is calculated from 1,000 time traces for each pulse energy after matched filtering. Only one in every 40 points for each energy is shown for clarity. The solid lines exhibit fits of the error function to the experimental data. **b**, Mean values divided by FWHMs extracted from fitting the distributions shown in **a**, as functions of the pulse energy (crosses). Intermediate values (line) are obtained by interpolating the fit parameters. The energy resolution is the energy at which the mean signal is equal to the FWHM, indicated by the vertical dashed line. The error bars and shaded regions denote 1-standard-deviation confidence intervals for the fitted and interpolated data, respectively.

Next, we construct empirical cumulative distribution functions (CDFs) of the calorimetric signals $\bar{S}$ from 1,000 traces for each pulse energy. We find that the noise in the signal is well approximated by a normal distribution, and we thus obtain a good agreement with fits of the CDFs to the error function, as shown in Fig. 4a. From these fits, we extract the means ${\mu }_{\bar{S}}$ and standard deviations ${\sigma }_{\bar{S}}$ of the distributions for each pulse energy. We convert these to the mean *μ*_*E*_ and standard deviation *σ*_*E*_ of the energy measured by the calorimeter using the calibrated energies of the pulses reaching the chip, and a model for the dependence of the signal on the pulse energy (Methods).

With large pulse energies *E*_MW_, the resonance frequency of the tank circuit moves far away from the probe frequency, and thus the probe signal effectively saturates. This is reflected in the widths of the distributions of the measured energy increasing considerably in Fig. 4a. Similarly, at zero pulse energy, our chosen probe frequency lies near the peak of the Lorentzian transmission of the sensor output (equation (4)), which results in a comparably large *σ*_*E*_ even though ${\sigma }_{\bar{S}}$ does not vary considerably with the pulse energy (Methods).

**Extended Data Fig. 3:**
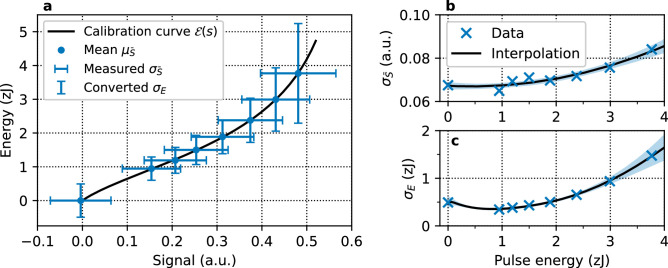
Conversion between the measured calorimetric signal and the energy at the input of the sensor. **a**, Mean energy in the input pulses of the calorimeter as a function of the mean value ${\mu }_{\bar{S}}$ of the normal distribution of the corresponding measured signal distribution for 1000 traces (dots). Conversion of arbitrary signal level to energy units is obtained from the calibration curve ${\mathcal{E}}(s)$ (solid line). The horizontal error bars denote the fitted standard deviation ${\sigma }_{\bar{S}}$ in signal units, and the vertical bars the corresponding uncertainty in energy *σ*_*E*_, obtained by transforming the variance by ${\mathcal{E}}$ (see text). **b**, Fitted standard deviation ${\sigma }_{\bar{S}}$ in signal units (crosses) as a function of the calibrated pulse energy. The solid line shows a quadratic polynomial fit that is used to interpolate the values of ${\sigma }_{\bar{S}}$. **c**, Standard deviation (crosses) and the interpolated curve (solid line) after transforming the data of **b** using ${\mathcal{E}}$. The shaded regions in panels **b** and **c** indicate the one-standard-deviation confidence intervals *δσ*_*s*_ and *δσ*_*E*_, respectively.

Note that fluctuations in the resonance frequency, such as those owing to thermal fluctuations in the thermometer or the Poissonian distribution of photons in the input pulse^44^, should result in a distribution for $\bar{S}$ that is distorted by the Lorentzian dependence of the signal on the resonance frequency. This effect should be especially pronounced for low pulse energies and *f*_p_ chosen close to the resonance, as is our case. Yet, we find the signal to very closely follow a normal distribution. Furthermore, at high powers, fluctuations in the signal $\bar{S}$ owing to fluctuations in the resonance frequency should reduce as a result of the aforementioned saturation. This is the opposite of what we find for ${\sigma }_{\bar{S}}$ before conversion to energy (Methods and Extended Data Fig. 3b), as it increases with the signal power. The noise in the output of the sensor is a combination of several components^21,45^. Compared with, for example, TESs, the noise sources of which have been well studied^24,46^, a thorough analysis of the noise in SNS sensors is lacking in the literature (see ref. ^47^ for a limited analysis), and hence the relative contributions of the different sources are challenging to distinguish on the basis of the available data.

Fundamentally, the energy resolution of any thermal detector is limited by the thermodynamic fluctuations in its absorber and thermometer elements. The order of magnitude of the energy resolution limit owing to these fluctuations can be estimated at low frequencies as^45^ $\Delta {E}_{{\rm{th}}} \sim 2\sqrt{2\ln 2{k}_{{\rm{B}}}{T}_{{\rm{e}}}^{2}{C}_{{\rm{e}}}}$, in the absence of feedback^48^. Here *k*_B_ is the Boltzmann constant and *C*_e_ is the absorber heat capacity, which is in our case that of the nanowire electrons. On the basis of the free electron model, the electronic heat capacity is given by *C*_e_ = *γVT*_e_, where *γ* ≈ 62 J K^−2^ m^−3^ for AuPd^49^ and *V* ≈ 3.6 μm × 150 nm × 30 nm is the volume of the nanowire not covered by Al, including both the absorber and thermometer segments. Assuming a steady-state electron temperature of *T*_e_ = 60 mK, this predicts *ΔE*_th_ ≈ 0.13 zJ. This agrees well with ref. ^39^, where this limit was estimated to be approximately 0.28 zJ for a device similar to ours. Comparing this to the realized energy resolution below, we expect the sensitivity to be at least partially limited by the amplification chain and not fully by the sensor itself. This expectation is consistent with the results of ref. ^33^, where placing a quantum-limited amplifier directly at the probe output at the millikelvin stage of the cryostat improved the NEP roughly by a factor of two and correspondingly the estimated energy resolution. Furthermore, we note that *ΔE*_th_ can be considerably lower than $2\sqrt{2{\rm{ln}}2{k}_{{\rm{B}}}{T}_{{\rm{e}}}^{2}{C}_{{\rm{e}}}}$ if the internal thermalization of the sensor is fast and it is being measured over such a broad band^21,45^.

We determine the energy resolution of our calorimeter by the input energy that yields a signal-to-FWHM ratio of unity, that is, resolving power of unity, as shown in Fig. 4b. Although no data with a pulse energy below *E*_MW_ = 0.95 zJ is available, the signal-to-FWHM ratio with (0.95 ± 0.02) zJ pulse energy is 1.17 ± 0.05 > 1, which thus implies that the energy resolution of the calorimeter is finer than (0.95 ± 0.02) zJ. This corresponds to approximately 171 ± 4 photons at the microwave signal frequency of 8.40 GHz. The uncertainty of 0.02 zJ or 4 photons arises from an uncertainty of 0.1 dB in the calibration of the input line attenuation (Methods). By interpolating *σ*_*E*_ (Methods), we estimate the actual energy resolution of the calorimeter to be (0.83 ± 0.04) zJ, corresponding to approximately 150 ± 7 photons. This agrees reasonably well with the energy resolution of 1.03 zJ predicted by the NEP.

Owing to the large shift of the resonance, the signal-to-FWHM ratio reaches a maximum near *E*_MW_ = 2 zJ and drops below unity at higher energies, signifying a dynamic range of only a few zeptojoules. This could be mitigated, for example, by using a frequency comb or simply a second probe tone at a frequency near the shifted resonance, but in this work, we have focused on optimizing the energy resolution instead of the dynamic range.

## Conclusions

We have reported a radiation sensor based on SNS junctions operated in a calorimetric mode. At our chosen operation point, the thermal time constant *τ* = 260 μs and ${\rm{NEP}}=49\,{\rm{zW}}/\sqrt{{\rm{Hz}}}$ of the device are on par with the state-of-the-art values for ultrasensitive bolometers. We have measured the FWHM energy resolution to be finer than 0.95 zJ = 5.9 meV when detecting 8.4 GHz microwave signals. By interpolating our data, we further estimate that the energy resolution may be as fine as 0.83 zJ = 5.2 meV.

An important factor in reaching this result was the use of a matched filter in the processing of the SNS sensor data, which improved the SNR by more than 30% compared with simple averaging in the time domain. Our results indicate that a considerable source of noise in the measured signal is the amplification chain after our device, as has been reported previously for similar devices. A detailed characterization of the importance of different noise sources is left for future work, but on the basis of the results of ref. ^33^ we expect that the NEP, and thus the energy resolution, may be improved by a factor of approximately two by using a quantum-limited parametric amplifier at the millikelvin stage of the cryostat.

A further key direction for future research is a comprehensive analysis of how the device design affects the performance. For example, the reasonably low quality factor of around 120 may possibly be increased by reducing the number of SNS junctions, shortening the junctions, or lowering the probe frequency. This may improve the sensitivity since a similar shift in the resonance frequency would lead to a larger change in the reflection coefficient and, furthermore, the heat capacity of the nanowire electrons may be reduced. On the other hand, a narrower resonance may decrease the speed of the sensor due to a smaller rate of photon emission from the probe circuit. Theoretically predicting the energy resolution is challenging since it depends on several interrelated factors. These include the thermal dynamics of the nanowire, the material properties of AuPd, the contacts between the superconductor and the normal metal, the speed at which the resonance shifts, the rate of photon emission from the probe circuit and the electrothermal feedback. Therefore, optimizing the device design calls for extended studies.

We expect that using similar data analysis methods, SNS sensors based on absorbers with lower heat capacity, such as graphene, could further improve the energy resolution. In the future, we aim to pursue this experiment together with a demonstration of the case where the arrival time of the signal to be detected is unknown, which is relevant for practical applications such as the detection of cosmic radiation. Our energy resolution corresponds to a single 1.4 THz photon. However, it should be noted that the absorption efficiency of AuPd at 1.4 THz and above is expected to be lower compared with that at microwave frequencies^50^. A reduction in the absorption of high-energy photons has been observed in TESs^32,51^, where it is attributed to the high-energy photon generating high-energy phonons, which may escape the absorber without heating the electrons. The existence of this effect should be investigated in AuPd SNS sensors.

A radiation sensor with sub-zeptojoule energy resolution is of interest for a broad range of applications, including astrophysics and cosmology, quantum technology, and quantum thermometry. Our work lays the foundation for such applications and eventually for calorimetry down to the single microwave-photon level. Immediate advantages may be obtained by adapting the matched-filtering scheme of this work to the calorimetric readout of superconducting qubits^40^.

## Methods

### Full experimental setup

A detailed depiction of the experimental setup is shown in Extended Data Fig. 1. A NI 5782R transceiver module sent digital triggers to apply pulse modulation to a PXIe-5654 microwave source to generate the microwave pulses.

The continuous probe tone was generated by another NI PXIe-5654 microwave source. The signal was split using a directional coupler into the probe signal entering the cryostat and a reference signal. The probe tone was attenuated and filtered, and reflected off of the SNS sensor via a directional coupler. After being reflected, the signal was amplified by a Miteq high-electron-mobility-transistor amplifier at the 4 K stage of the cryostat, and then by room-temperature low-noise amplifiers. The noise temperature of the high-electron-mobility-transistor amplifier was not specified by the manufacturer at 4 K, but at 77 K it was specified to be 58.7 K with a reference temperature of 290 K. Thus, we expected the noise temperature of the high-electron-mobility transistor amplifier to be less than this at the 4 K operating temperature.

The probe signal was demodulated from radio frequency by mixing it with a local oscillator signal, which was always detuned from *f*_p_ by the fixed intermediate frequency 70.3125 MHz. After demodulation, the signal was further filtered to remove sidebands from the mixing, and amplified more at the intermediate frequency.

The probe signal was digitized by the NI 5782R at a sampling rate of 250 MSa s^−1^ and digitally demodulated from the intermediate frequency to d.c., yielding the heterodyne in-phase (*I*) and quadrature-phase (*Q*) components. Finally, the signal was averaged and decimated over 2^7^ adjacent samples for a digital time step of 512 ns. The reference signal was directly demodulated and digitized, and it was used as a phase reference for the digitized probe tone.

Attenuators were placed between amplifiers to avoid standing waves from reflections between the components. The 1-to-6 switches in Extended Data Fig. 1 were used to switch between the sample used in our experiment and other samples unrelated to this work.

The total input line attenuation was calibrated following the procedure introduced in ref. ^52^. In a separate thermal cycle of the cryostat, we replaced the SNS sensor used in the main experiment with a bolometer that could be heated both with microwave power and d.c. current. We applied a known d.c. power to the nanowire using a four-wire configuration, and by varying the radio frequency powers applied at room temperature and comparing the resulting frequency shift, we found correspondence between the room-temperature radio frequency power and the power at the chip. We found that the total input line attenuation was 119.24 ± 0.1 dB. Note that this was the total attenuation up to the input of the sample holder containing the chip used in the main experiment. Thus the reported energy resolution considered our sensor to be a black box at the end of a 50-Ω transmission line, and it included possible reflections owing to impedance mismatch at the chip input that degraded the measured energy resolution. We considered the change in line attenuation between the thermal cycles of the cryostat to be negligible.

### Model for bolometric transmission

In the bolometric linear mode of operation, the steady-state reflection coefficient of the gate capacitor at a given probe frequency *f*_p_ follows a Lorentzian line shape^53^:

1 $$\Gamma \propto 1-\frac{{{\rm{e}}}^{{\rm{i}}\varphi }{\gamma }_{{\rm{c}}}}{\gamma /2+2\pi {\rm{i}}\left(\,{f}_{{\rm{r}}}-{f}_{{\rm{p}}}\right)},$$

where *f*_r_ is the resonance frequency of the LC tank circuit of the sensor, *γ*_c_ and *γ* are the external and total energy decay rates, respectively, and *φ* is a parameter corresponding to the asymmetry of the resonance owing to impedance mismatch between the chip and the coaxial cable at the probe input. The resonance frequency shifts approximately linearly with the power *P*_MW_ of the microwave tone^44^ (or equivalently, the pulse energy *E*_MW_ = *P*_MW_ *t*_MW_):

2 $${f}_{{\rm{r}}}={f}_{{\rm{r}},0}-\alpha {P}_{{\rm{MW}}},$$

where *f*_r,0_ is the resonance frequency with no microwave pulse, and *α* is a coefficient with units of Hz W^−1^ corresponding to the conversion of *P*_MW_ to heat in the absorber, and subsequently to a change in the Josephson inductance of the nanowire. In general, *α* depends on several factors, including the absorber geometry and material, and impedance matching of the absorber to the input coaxial line.

The difference in the complex heterodyne transmitted signal *I* + i*Q* with a given input power is thus proportional to

3 $$\begin{array}{l}\Gamma ({\rm{P}}={{\rm{P}}}_{\mathrm{MW}})-\Gamma ({\rm{P}}=0)\\ \propto \left(\frac{1}{{\rm{\gamma }}/2+2{\pi i}\left({{f}}_{{\rm{r}}\mathrm{,0}}-{{f}}_{{\rm{p}}}\right)}-\frac{1}{{\rm{\gamma }}/2+2{\pi i}\left({{f}}_{{\rm{r}}\mathrm{,0}}-{\mathrm{\alpha P}}_{\mathrm{MW}}-{{f}}_{{\rm{p}}}\right)}\right){{\rm{e}}}^{{i\varphi }}{{\rm{\gamma }}}_{{\rm{c}}}.\end{array}\,\,\,\,$$

The relative change in the digitized voltage Δ*V* is obtained by applying a rotation to cancel out *φ*, multiplying by the total gain of the amplification chain *G*, and discarding the imaginary part as

4 $$\begin{array}{rcl}\Delta V & = & G{\gamma }_{{\rm{c}}}\,\mathrm{Re}\,\left[\frac{1}{\gamma /2+2\pi {\rm{i}}\left({f}_{\mathrm{r,0}}-{f}_{{\rm{p}}}\right)}-\frac{1}{\gamma /2+2\pi {\rm{i}}\left(\,{f}_{\mathrm{r,0}}-\alpha {P}_{\mathrm{MW}}-{f}_{{\rm{p}}}\right)}\right]\\ & = & G\frac{\gamma {\gamma }_{{\rm{c}}}}{2}\left(\frac{1}{{(\gamma /2)}^{2}+{\left[2\pi \left({f}_{{\rm{r}},0}-{f}_{{\rm{p}}}\right)\right]}^{2}}-\frac{1}{{(\gamma /2)}^{2}+{\left[2\pi \left(\,{f}_{{\rm{r}},0}-\alpha {P}_{\mathrm{MW}}-{f}_{{\rm{p}}}\right)\right]}^{2}}\right).\end{array}$$

With large values of *P*_MW_, this expression becomes effectively constant, and hence the energy resolution of the sensor approaches zero. However, it may still be used as a binary detector, indicating the presence or absence of a microwave signal.

### Estimation of the noise PSD

The one-sided noise PSD *S*_n_ of the measured voltage is used both in calculating the NEP and in the matched-filtering procedure. We estimate the noise PSD by averaging periodograms according to Bartlett’s method^54^ for data with no pulse applied.

The PSD used in the matched filtering is obtained by fitting the estimated PSD to a simple heuristic model ${\widetilde{S}}_{{\rm{n}}}(f)=A/f+B$, which is a sum of 1/*f* and white noise. Using this model instead of the estimated PSD directly yields a better SNR, since the estimate is noisy (Extended Data Fig. 2a). The measured spectrum does not completely agree with this model, which we attribute to the analogue filtering and amplification in the output chain. Nevertheless, we find that using the model in the matched-filtering procedure improves the energy resolution by approximately 4% compared with using the raw noise PSD. Note that one may freely choose the noise model in the matched-filtering procedure without loss of scientific soundness.

### Obtaining the NEP

To obtain the NEP from the experimental data in the bolometric mode, we record time-domain traces of the transmitted signal similar to the one shown in Fig. 1d for different values of *f*_p_ and *P*_p_. We offset the time axis such that *t* = 0 is at the arrival time of the pulse, and fit these traces to the model $\Delta V\times (1-{{\rm{e}}}^{-t/\tau })$ for *t* > 0 to extract the relative-voltage signal *ΔV* and thermal time constant *τ*.

With *f*_p_ close to *f*_r,0_ and *αP*_MW_ ≪ *γ*, we have an approximately linear dependence of Δ*V* on *P*_MW_, and hence we define the quasistatic responsivity as *δV*/*δP*_MW_ = Δ*V*/*P*_MW_, where the word quasistatic refers to the fact that this definition assumes that *P*_MW_ is constant in time. To determine how the responsivity varies with temporal changes in *P*_MW_ occurring at the noise frequency *f*_n_, the responsivity may be measured while modulating *P*_MW_ at *f*_n_. However, we capture the frequency dependence of the responsivity by^21,33,55^ $\delta V/\delta {P}_{\mathrm{MW}}{[1+{(2\pi {f}_{{\rm{n}}}\tau )}^{2}]}^{-1/2}$, which takes into account the decrease in responsivity owing to the thermal time constant *τ*. Note that this procedure is justified by our empirical observation in Fig. 1d that the rise of the sensor signal for *P*_MW_ suddenly turned on is accurately exponential.

The NEP is thus given by

5 $$\,\mathrm{NEP}\,\left(\,{f}_{{\rm{n}}}\right)=\sqrt{{S}_{{\rm{n}}}\left(\,{f}_{{\rm{n}}}\right)}{\left(\delta V/\delta {P}_{\mathrm{MW}}\right)}^{-1}\sqrt{1+{\left(2\pi {f}_{{\rm{n}}}\tau \right)}^{2}},$$

where *S*_n_(*f*_n_) is the noise PSD of the measured relative voltage Δ*V* at the frequency *f*_n_. The units of the NEP are ${\rm{zW}}/\sqrt{{\rm{Hz}}}$ since it equals the square root of the noise PSD of the measured signal in units of input power to the bolometer. The NEP obtained at the operation point used for the calorimetry is shown in Extended Data Fig. 2b.

For the probe parameters considered in Fig. 2, the NEP is lowest around the range of 0.3–1 kHz, indicated by the shaded region in Extended Data Fig. 2b. In this frequency range, the effect of the 1/*f* noise is reduced, but the insensitivity owing to finite *τ* is not yet considerable. This implies that the highest sensitivity in the bolometric mode can be achieved by modulating the input power *P*_MW_ at a frequency within this range, for example, by using a shutter, a multiplexer that switches between two bolometers, or by using lock-in detection. Thus we choose to average over this range for the data shown in Fig. 2c.

The NEP also allows us to estimate the energy resolution, through the relation^21^

6 $$\Delta {E}_{\mathrm{FWHM}}=2\sqrt{2\ln 2}{\left({\int }_{0}^{{f}_{\max }}\frac{4}{{\mathrm{NEP}}^{2}(\,f\,)}{\rm{d}}f\right)}^{-1/2},$$

where the factor $2\sqrt{2\ln 2}$ arises from the used FWHM of a normally distributed signal (discussed below) and ${f}_{\max }$ is a frequency, up to which the sensor is used. In our previous work^33^, we have used the thermal cut-off 1/(2*πτ*) for the upper bound of the integral, but we find that in our case, this gives an unnecessarily pessimistic estimate. This equation is used to obtain the data shown in Fig. 2d.

### Single-shot data processing using matched filtering

Here we discuss the data processing used for the data in the calorimetry experiments where no ensemble-averaging is carried out. The phase of each digitized and down-converted heterodyne trace relative to each other is normalized using a phase reference (Extended Data Fig. 1), and the baseline is removed by subtracting the median of the *I* and *Q* components of each trace for the section before the pulse. The baseline-removed traces are rotated in the *I*–*Q* plane by a global phase angle $\widetilde{\varphi }$ such that projecting the rotated signal to the *I* axis yields the finest energy resolution. An example of such a digitized trace after this preprocessing step is shown in Fig. 3a.

After the preprocessing, a matched filter^21,43,56^ is applied to the projected in-phase signal. The matched filter is the linear time-invariant filter that maximizes the SNR, and hence it is sometimes called an optimal filter. For the matched filter, the filtered signal is given by the following convolution:

7 $${S}_{k}={\mathrm{DFT}}^{-1}{\left[\frac{\mathrm{DFT}{\left[{V}_{k}\right]}_{j}\times {\overline{\mathrm{DFT}\left[{K}_{k}\right]}}_{j}}{{S}_{{\rm{n}},\,j}}\right]}_{k},$$

where *V*_*k*_ is the digitized signal at the sample index *k*, *K*_*k*_ is the template of the matched filter (discussed below), $\overline{z}$ denotes the complex conjugate of *z*, ${S}_{{\rm{n}},j}={S}_{{\rm{n}}}\left({f}_{{\rm{n}},j}\right)$ is the noise PSD at the frequency bin with index *j*, and DFT and DFT^−1^ denote the discrete Fourier transform and its inverse, respectively.

The values of the signal after the matched filtering, *S*_*k*_, indicate how well the raw signal and template correlate at a given convolution offset, with additional weighting from the noise PSD emphasizing frequency components that are less noisy. If the pulse arrival time is known and the template has a duration equal to the window of the digitized signal, the matched filter yields a peak at zero convolution offset. The final calorimetric signal $\bar{S}$ is obtained by taking the mean of the filtered signal *S*_*k*_ over a 1.024-μs window centred at zero convolution offset, as shown in Fig. 3c.

The template of the matched filter is the expected time-domain shape of the signal, which is assumed to be directly proportional to the energy of the pulse. For the short pulses used in the calorimetry, we model the temporal dependence of the projected signal as a sum of two exponentially decaying processes as

8 $$K(t)=\left\{\begin{array}{ll}0 & t < 0,\\ ({a}_{1}+{a}_{2})\times t/{t}_{{\rm{MW}}} & 0\le t < {t}_{{\rm{MW}}},\\ {a}_{1}\exp \left[-(t-{t}_{{\rm{MW}}})/{\tau }_{1}\right]+{a}_{2}\exp \left[-(t-{t}_{{\rm{MW}}})/{\tau }_{2}\right] & {t}_{{\rm{MW}}}\le t,\end{array}\right.$$

where the arrival time of the pulse is *t* = 0 and *t*_MW_ = 1 μs is the length of the pulse. The amplitude *a*_1_ and time constant *τ*_1_ correspond to a fast decay of heat from the electrons to an intermediate heat bath, and *a*_2_ and *τ*_2_ to a slow decay from the intermediate bath to the phonon bath of the chip substrate through a weak thermal link. The exact physical origin of this double-exponential behaviour is unknown, but such behaviour has been reported in earlier experiments with similar devices^39,57^. Here we assume a zero baseline, and that the length of the input pulse *t*_MW_ is much shorter than the time constants *τ*_1_ and *τ*_2_, so that the signal rises effectively linearly during the pulse. We extract the template by ensemble-averaging 1,000 pulses with a relatively high pulse energy of 3.8 zJ and fitting the data to equation (8). From the fit, we obtain *τ*_1_ ≈ 18 μs, *τ*_2_ ≈ 150 μs and *a*_2_/*a*_1_ ≈ 1.5. The template with these parameters is shown in Fig. 3b.

With high powers, the heterodyne signal moves along a curve in the *I*–*Q* plane, as predicted by equation (3). Thus the linear relationship between the pulse energy and the projected signal Δ*V* breaks down, and the effectiveness of the matched filter is reduced. While we observe some deviation from the linear behaviour in our data, we find that this effect does not considerably affect the filtering procedure for the pulse energies considered here.

### Conversion of filtered signal to energy units and calculation of the energy resolution

The calorimetric signal $\bar{S}$ extracted from the matched-filtering procedure has arbitrary units, since we have not calibrated the whole amplification chain from the sample to the analogue-to-digital converter. Here we discuss the procedure used to convert the extracted signal and its standard deviation to units of energy for Fig. 4, and how that is subsequently used to obtain the energy resolution.

For each pulse energy *E*_MW_, we collect *N* = 1,000 traces and extract the corresponding signals ${\bar{S}}_{j}$, *j* = 1, …, *N* as discussed above. We then compute the empirical CDF for each *E*_MW_, given by

9 $${\mathrm{CDF}}_{{E}_{\mathrm{MW}}}(s)=\mathop{\sum }\limits_{{\bar{S}}_{j}\le s}\frac{1}{N}.$$

The noise in the signal closely follows a normal distribution, and therefore we model the CDF for a given pulse energy *E* as

10 $${\mathrm{CDF}}_{E}(s)=\frac{1}{2}+\frac{1}{2}\mathrm{erf}\,\left(\frac{s-{\mu }_{\bar{S}}(E\,)}{\sqrt{2}{\sigma }_{\bar{S}}(E\,)}\right),$$

where erf( ⋅ ) is the error function, and ${\mu }_{\bar{S}}$ and ${\sigma}_{\bar{S}}$ are the mean and standard deviation of the corresponding normal distribution, respectively. We extract ${\mu }_{\bar{S}}$ and ${\sigma }_{\bar{S}}$ by a least-square fit of the error function in equation (10) to the empirical CDF.

Since we have calibrated the total attenuation at the input of the microwave absorber, the energy corresponding to each value of ${\mu }_{\bar{S}}$ is known, up to a constant relative error of ±0.1 dBm, discussed below. Since $\bar{S}$ is proportional to *δV*/*δP*_MW_, its dependence on *E*_MW_ is also of the form of equation (4) (with *P*_MW_ = *E*_MW_/*t*_MW_ and *α* adjusted). We invert equation (4) by solving for *E*_MW_ to obtain

11 $${\mathcal{E}}(s)=\frac{{t}_{{\rm{MW}}}}{2\pi \alpha }\left(2\pi \left({f}_{{\rm{r}},0}-{f}_{{\rm{p}}}\right)+\sqrt{{\left[\frac{1}{{(\gamma /2)}^{2}+{\left[2\pi \left({f}_{{\rm{r}},0}-{f}_{{\rm{p}}}\right)\right]}^{2}}-\frac{s}{a}\right]}^{-1}-1}\right),$$

where *a* = *Gγγ*_c_/2 is a scaling parameter of the measured signal corresponding to the unknown prefactor in equation (4). We fit this model to the extracted values of ${\mu }_{\bar{S}}$ with ${\widetilde{\Delta }}_{f}=({f}_{{\rm{r}},0}-{f}_{{\rm{p}}})/\alpha$, $\widetilde{a}=a/\alpha$, and $\widetilde{\gamma }=\gamma /\alpha$ as the fitting parameters. This then allows using ${\mathcal{E}}(s)$ as a calibration curve that maps a signal *s* to the corresponding energy reported by the calorimeter. Extended Data Fig. 3a shows ${\mu }_{\bar{S}}$ and ${\mathcal{E}}(s)$. To convert the standard deviation ${\sigma }_{\bar{S}}(E)$ at a given energy to the standard deviation *σ*_*E*_(*E*) that is in units of energy, we use the following relation^58^ for the variance of a random variable transformed by a nonlinear function:

12 $${\sigma }_{E}^{2}\approx {{\mathcal{E}}}^{2}({\mu }_{\bar{S}})+{\sigma }_{\bar{S}}^{2}\left({\left[{{\mathcal{E}}}^{{\prime} }({\mu }_{\bar{S}})\right]}^{2}+{\mathcal{E}}({\mu }_{\bar{S}}){{\mathcal{E}}}^{{\prime\prime} }({\mu }_{\bar{S}})\right)-{\left[{\mathcal{E}}({\mu }_{\bar{S}})+\frac{{\sigma }_{\bar{S}}^{2}}{2}{{\mathcal{E}}}^{{\prime\prime} }({\mu }_{\bar{S}})\right]}^{2}.$$

The standard deviations ${\sigma }_{\bar{S}}$ obtained using equation (10) and the corresponding *σ*_*E*_ are shown in Extended Data Fig. 3a as horizontal and vertical bars, respectively, as well as in Extended Data Fig. 3b,c as crosses. The FWHM is that of a Gaussian function, given by ${W}_{E}={\sigma }_{E}\times 2\sqrt{2\ln 2}$. The data points in Fig. 4b are thus calculated as ${E}_{{\rm{MW}}}/{W}_{{E}_{{\rm{MW}}}}$.

To estimate *σ*_*E*_ between the values we have measured, we approximate ${\sigma }_{\bar{S}}(E)$ by the quadratic polynomial *σ*_*s*_(*E*) = *a*_0_ + *a*_1_*E* + *a*_2_*E*^2^ that is fit to the measured ${\sigma }_{\bar{S}}$. We then transform this interpolated curve according to equation (12) (with $s={{\mathcal{E}}}^{-1}(E)$ in place of ${\mu }_{\bar{S}}$), resulting in the curve shown in Extended Data Fig. 3c. This yields the interpolated FWHM *W*(*E*), and subsequently the interpolated curve in Fig. 4b, which is given by *E*/*W*(*E*). Finally, the estimated energy resolution of 0.83 zJ is determined by numerically finding *E* such that *E* = *W*(*E*).

Note that the projection angle $\widetilde{\varphi }$, as well as the averaging window discussed above are chosen such that the energy resolution estimated with this method is optimized. Nevertheless, we find that for the 0.95 zJ pulse, even if $\widetilde{\varphi }$ is offset by up to 0.1 × 2*π* from its optimal value, the measured signal-to-FWHM ratio can be made greater than unity by adjusting the averaging window offset and length by 1–2 μs.

### Uncertainty of the energy resolution

Here we discuss how we obtain the uncertainties of 0.02 zJ in the pulse energy 0.95 zJ and 0.04 zJ in the energy resolution estimate. It is important to make a distinction between the standard deviations *σ*_*s*_ and *σ*_*E*_, which stem from noise in the signal and are obtained by fitting the empirical CDFs, and their uncertainties *δσ*_*s*_ and *δσ*_*E*_, respectively, which are derived from the fit. We use uncertainty to exclusively refer to *δσ*_*s*_ and *δσ*_*E*_, as well as to other sources of error such as the 0.1 dB of uncertainty in the calibration of the line attenuation.

We obtain the uncertainty in *E*/*W*(*E*) as follows:

13 $$\delta \left[\frac{E}{W(E)}\right]=\frac{E}{W(E)}\sqrt{{\left(\frac{\delta E}{E}\right)}^{2}+{\left(\frac{\delta W(E)}{W(E)}\right)}^{2}},$$

where *δE* is given by 0.1 dB × *E* ≈ 1.023 × *E*, and

14 $$\delta W(E)=2\sqrt{2\ln 2}\,\delta {\sigma }_{E}(E)=2\sqrt{2\ln 2}\,{\sigma }_{s}(E){{\mathcal{E}}}^{{\prime} }(s)\sqrt{{\left(\frac{\delta {\sigma }_{s}(E)}{{\sigma }_{s}(E)}\right)}^{2}+{\left(\frac{\delta {{\mathcal{E}}}^{{\prime} }(s)}{{{\mathcal{E}}}^{{\prime} }(s)}\right)}^{2}},$$

where

15 $$\delta {\sigma }_{s}(E)={\left[{\nabla }_{{\rm{P}}}{\sigma }_{s}(E)\right]}^{{\rm{T}}}{{\bf{C}}}_{{\sigma }_{s}}{\nabla }_{{\rm{P}}}{\sigma }_{s}(E),$$

and

16 $$\delta {{\mathcal{E}}}^{{\prime} }(s)={\left[{\nabla }_{{\rm{P}}}{{\mathcal{E}}}^{{\prime} }(s)\right]}^{{\rm{T}}}{{\bf{C}}}_{{{\mathcal{E}}}^{{\prime} }}{\nabla }_{{\rm{P}}}{{\mathcal{E}}}^{{\prime} }(s),$$

where $s={{\mathcal{E}}}^{-1}(E)$ and ∇_P_*σ*_*s*_(*E*) denotes the gradient of *σ*_*s*_(*E*) with respect to each of its parameters: ${\nabla }_{{\rm{P}}}{\sigma }_{s}(E)={[{\partial }_{{a}_{0}}{\sigma }_{s}(E),{\partial }_{{a}_{1}}{\sigma }_{s}(E),{\partial }_{{a}_{2}}{\sigma }_{s}(E)]}^{{\rm{T}}}$, and similarly ${\nabla }_{{\rm{P}}}{{\mathcal{E}}}^{{\prime} }(s)$ denotes the gradient of ${{\mathcal{E}}}^{{\prime} }(s)$ with respect to the parameters of the Lorentzian: ${\nabla }_{{\rm{P}}}{{\mathcal{E}}}^{{\prime} }(s)={[{\partial }_{\widetilde{a}}{{\mathcal{E}}}^{{\prime} }(s),{\partial }_{\widetilde{\gamma }}{{\mathcal{E}}}^{{\prime} }(s),{\partial }_{{\widetilde{\Delta }}_{f}}{{\mathcal{E}}}^{{\prime} }(s)]}^{{\rm{T}}}$, and ${{\bf{C}}}_{{\sigma }_{s}}$ and ${{\bf{C}}}_{{{\mathcal{E}}}^{{\prime} }}$ are the covariance matrices of the parameters, obtained from the fitting procedure described above. We have included the uncertainties in ${\sigma }_{\bar{S}}$ and ${\mu }_{\bar{S}}$ obtained from the CDF fitting as weights in the fitting of ${\mathcal{E}}$ and *σ*_*s*_, so that the effect of these are included in the covariance matrices.

Note also that the Poissonian statistics of the coherent input photons cause fluctuations in the input photon number with a standard deviation of $\sqrt{E/(h{f}_{{\rm{MW}}})}$, or equivalently, fluctuations in the energy with a standard deviation of $\sqrt{Eh{f}_{{\rm{MW}}}}$. The effect of these fluctuations is already included in ${\sigma }_{\bar{S}}$, and hence also in the signal-to-FWHM ratio we have used to determine the energy resolution. For the pulse energies we have measured, this corresponds to 13–26 photons or 0.07–0.14 zJ.

The uncertainty obtained from equation (13) is used to calculate the error bars and the confidence interval shown in Fig. 4b, and *δσ*_*s*_ and *δσ*_*E*_ are shown as the error bars and confidence intervals in Extended Data Fig. 3b,c, respectively. Using these confidence intervals, we find the points where *E*/*W*(*E*) ± *δ*[*E*/*W*(*E*)] = 1, which yields (0.83 ± 0.04) zJ. We note that the uncertainty is dominated by *δσ*_*s*_ and $\delta {{\mathcal{E}}}^{{\prime} }$ since the relative uncertainties resulting from the CDF fit are negligible.

## Data Availability

The data that support the findings of this study are available via Zenodo at 10.5281/zenodo.16038651 (ref. ^59^).

## Code availability

The code that supports the findings of this study is available via Zenodo at 10.5281/zenodo.16038651 (ref. ^59^).
